# Baicalein Attenuates Brain Iron Accumulation through Protecting Aconitase 1 from Oxidative Stress in Rotenone-Induced Parkinson’s Disease in Rats

**DOI:** 10.3390/antiox12010012

**Published:** 2022-12-21

**Authors:** Run-Zhe Liu, Sen Zhang, Wen Zhang, Xiao-Yue Zhao, Guan-Hua Du

**Affiliations:** 1 State Key Laboratory of Bioactive Substances and Functions of Natural Medicines, Institute of Materia Medica, Chinese Academy of Medical Sciences and Peking Union Medical College, Beijing 100050, China; 2 Beijing Key Laboratory of Drug Target Identification and Drug Screening, Institute of Materia Medica, Chinese Academy of Medical Sciences and Peking Union Medical College, Beijing 100050, China; 3 Medical Science Research Center, Peking Union Medical College Hospital, Chinese Academy of Medical Science and Peking Union Medical College, Beijing 100730, China

**Keywords:** baicalein, Aconitase 1 (ACO1), iron regulatory protein 1 (IRP1), Parkinson’s disease, iron accumulation

## Abstract

Aconitase 1 (ACO1) links oxidative stress and iron accumulation in Parkinson’s disease (PD). ACO1 loses its aconitase activity and turns into iron regulatory protein 1 (IRP1) upon oxidative stress. IRP1 plays an important role in the accumulation of intracellular iron. Baicalein is a flavonoid isolated from the roots of Scutellaria baicalensis. The present results show that baicalein could bind to ACO1 and protect its isoform from the oxidative stress induced by reactive oxygen species (ROS) and reactive nitrogen species (RNS). Furthermore, baicalein promoted aconitase activity and inhibited IRP1 activation in rotenone-induced PD models. Additionally, baicalein decreased the hydroxyl radicals generated by iron. In conclusion, baicalein attenuated iron accumulation and iron-induced oxidative stress in the brain of PD rats by protecting ACO1.

## 1. Introduction

Parkinson’s disease (PD) is the second most common neurodegenerative disease worldwide. The loss of dopaminergic neurons and the accumulation of α-synuclein in the substantia nigra, are the neuropathological hallmarks of PD [1]. Oxidative stress, mitochondrial dysfunction, cellular calcium imbalance, neuroinflammation, and the imbalance of neurotransmitters are involved in the pathological mechanisms of PD [2]. The number of patients with PD is expected to double in the next 20 years, with more than 14 million patients worldwide by 2040 [3]. However, current PD therapies are limited to symptomatic treatment rather than disease-modifying interventions.

Iron accumulation in the substantia nigra is positively correlated with the severity of PD [4,5,6,7,8]. Furthermore, iron overload is reported in PD models induced by rotenone, 6-hydroxydopamine (6-OHDA), or 1-methyl-4-phenyl-1,2,3,6-tetrahydropyridine (MPTP) [9,10,11,12,13,14]. Alleviating iron accumulation is a potential therapeutic strategy for PD. Deferiprone is an iron chelator that removes excess iron from cells, improves dyskinesia, and attenuates iron accumulation in patients with PD [15].

Aconitase 1 (ACO1) is a key regulator of intracellular iron. Hydrogen peroxide or nitric oxide disrupts the iron–sulfur cluster of ACO1 and activates its iron regulatory protein 1 (IRP1) conformation [16,17]. IRP1 regulates the expression of TfR1, DMT1, and FPN1, to promote intracellular iron accumulation [18].

Baicalein (5,6,7-trihydroxyflavone) is a bioactive flavonoid isolated from the root of *Scutellaria baicalensis*. Baicalein demonstrates various biological activities and improves motor and non-motor symptoms in PD [19,20,21,22,23,24,25,26]. Baicalein has also been reported to chelate iron and alleviate iron accumulation [27,28]. Baicalein is well tolerated and shows little toxicity in humans [29]. Additionally, quercetin, an analog of baicalein, has recently been reported to antagonize the inhibition of mammalian aconitase by hydrogen peroxide [30]. This study aims to investigate the effects of baicalein on ACO1 through molecular, cellular, and in vivo experiments. The potential mechanisms wherein baicalein inhibits the activation of IRP1 are further studied.

## 2. Materials and Methods

### 2.1. Analysis of Mechanisms that Baicalein Regulates Intracellular Iron

The mechanisms of baicalein regulating intracellular iron were analyzed using Drug Regulation Path Searcher version 1.0.0, software developed by our laboratory earlier. The reported mechanisms of baicalein were collected from the literature and imported into the software. The maximum distance for pathway searching was set at 2. The results were visualized using Cytoscape version 3.8.2.

The molecular docking of baicalein to ACO1 was performed using AutoDock Vina version 1.2.0 (Scripps Research Institute, La Jolla, USA). The protein file (PDB:2B3X) was preprocessed with AutoDock Tools, i.e., polar hydrogens were added, and the pdbqt format was exported for molecular docking. The 3D structure of baicalein was obtained from the PubChem database.

### 2.2. Chemicals, Cells, and Animals

Baicalein was prepared by the Institute of Materia Medica, Chinese Academy of Medical Sciences & Peking Union Medical College. The purity of baicalein was >98%. Rotenone, sodium nitroprusside, and ferrous sulfate were purchased from Sigma-Aldrich (St. Louis, MO, USA). Fetal bovine serum (FBS) and Dulbecco’s modified Eagle’s medium (DMEM) were purchased from Gibco (Gaithersburg, MD, USA). Antibodies against ACO1, DMT1, TfR1, FPN1, and β-actin were purchased from Santa Cruz Biotechnology (Dallas, TX, USA). Goat anti-mouse/rabbit antibodies were purchased from CWBiotech (Beijing, China). Recombinant human ACO1 protein was purchased from Sino Biological (Beijing, China). The enzyme-linked immunosorbent assay (ELISA) kits for rat tetrahydrobiopterin (BH_4_) and coenzyme Q10 (CoQ_10_) were purchased from Jianglai Biotechnology (Shanghai, China). The other reagents were of commercially available analytical grade.

Human neuroblastoma SH-SY5Y cells were obtained from the Chinese Academy of Medical Sciences & Peking Union Medical College. The cells were grown as monolayers in DMEM with 10% heat-inactivated FBS and 5 μM ferrous sulfate at 37 °C in a humidified incubator with 95% air and 5% CO_2_. The cells in the exponential growth phase were treated with different concentrations of baicalein and 1 μM rotenone. Then, the cells were incubated for 24 h before further experiments.

Male SD rats, weighing 220 g, were purchased from Beijing Vital River Laboratory Animal Technology Co., Ltd. (Beijing, China). The animals were housed in an SPF environment with a 12 h/12 h light–dark cycle and had free access to food and water. All of the experiments were approved by the Ethics Review Committee for Animal Experimentation of the Institute of Materia Medica, Chinese Academy of Medical Sciences & Peking Union Medical College, and were in line with the requirements of the National Institutes of Health Guidelines for the Care and Use of Laboratory Animals (NIH Publications No. 8023, revised 1978).

### 2.3. Aconitase Activity Assay

The freshly collected rat hearts were washed in physiological saline to remove the blood and transferred into Tris-HCl buffer (20 mM, pH = 7.4). The mixture was homogenized and centrifuged at 12,000× *g* for 10 min. The aconitase activity was measured using a method described earlier with some modifications [31]. Briefly, the increase in aconitate absorbance in 240 nm was measured in Tris-HCl buffer, with 4 micromolar citrate and 100 μg of protein at room temperature. The relative aconitase activity was calculated using the following equation:

Activity (%) = (A_t20_ − A_t0_)/(A_u20_ − A_u0_) × 100% (1)

where A_t20_, A_t0_, A_u20_, and A_u0_ were the optical density (OD) of the sample at 20 min, sample at 0 min, uninhibited control at 20 min, and uninhibited control at 0 min, respectively.

### 2.4. Surface Plasmon Resonance (SPR) Assay

The interaction between baicalein and ACO1 was analyzed with a Biacore™ ×100 system (Chicago, IL, USA). In brief, the purified protein solution was adjusted to pH 4.0 with acetic acid and then coupled with a CM5 chip. Different concentrations of baicalein (100, 30, 10, 3, 1, and 0.3 μM) were diluted with a running buffer, and the samples were loaded to detect the response units (RU). Biacore ×100 evaluation software version 2.0.2 (Chicago, IL, USA) was used to analyze the data and calculate the Kd values.

### 2.5. Iron-Induced Hydroxyl Radical Assay

Hydroxyl radicals were captured by benzoic acid to produce hydroxybenzoic acid, which can be analyzed using fluorescence detection (λ_ex_ = 300 nm; λ_em_ = 408 nm) [32].

Baicalein, deferiprone, or levodopa (10 μM) were mixed with ferrous sulfate (50 μM) in PBS buffer (20 mM, pH = 7.4) and left incubating for 30 min. Then, ascorbic acid (2 mM) and benzoic acid (10 mM) were added (final concentrations shown in parenthesis). For the Fenton reaction, hydrogen peroxide (2 mM) was then added, and the fluorescence of the complex was measured immediately for 20 min. For iron and the dissolved-oxygen-induced hydroxyl radicals, no hydrogen peroxide was added, and the fluorescence of the complex was monitored for 24 h.

### 2.6. Animal Experiments

The rats were randomly divided into 5 groups: the control group, model group, BAI-300 group, BAI-150 group, and DFP-150 group. All of the groups received an intraperitoneal injection of 2.5 mg/kg rotenone q.d. for 6 weeks, except for the control group, which received an equal volume of vehicle. From the third week to the sixth week, the rats were administered 300 mg/kg of baicalein (BAI-300 group), 150 mg/kg baicalein (BAI-150 group), or 150 mg/kg of deferiprone (DFP-150 group) q.d. by gavage, while the control group and the model group received an equal volume of vehicle. The dose of baicalein was determined based on a previous study [20]; the dose of deferiprone was based on its clinical trial (30 mg/kg/d) and preclinical studies [10,15].

### 2.7. Behavioral Test

The rotarod performance test was conducted using the methods described by Sindhu et al. [33]. The rats were trained once a day for 3 days before the start of modeling. The test time was set on days 0, 14, 21, 28, 35, and 42.

To perform an inclined plane test, the rats were placed on a rough surface at an angle of 60 degrees to the ground with rubber barbs. The rats were placed in the center of the rough surface, and the retention time on the inclined plate was recorded, with an upper limit of 120 s. Three tests were performed, each with a 30 min interval. The rats were trained once a day for 3 days prior to the start of modeling. The test was performed on days 0, 14, 21, 28, 35, and 42.

A forced-swimming test was conducted using the methods described by Porsult et al. with some modifications [34]. Briefly, the rats were put into a water-filled glass tank with a water depth of 50 cm and at a temperature of 25 °C. The rat’s tail and hind limbs could not touch the bottom to support the body, nor could they climb out of the tank. After the rats entered the water, their immobility time was recorded. A forced-swimming test was carried out on day 43.

### 2.8. Immunochemistry

The brain tissue samples were collected from 3 rats in each group at random after the final behavioral test. The rats were anesthetized and perfused with 4% paraformaldehyde. Then, the brains were removed and fixed in 4% paraformaldehyde at 4°C overnight. Then, 4-μM-thick coronal sections were prepared at Bregma 0.6 mm (striatum) and Bregma −4.92 mm (substantia nigra). The sections were then subjected to tyrosine hydroxylase (TH) immunohistochemical staining.

### 2.9. Sample Preparation

The cell samples were lysed with 0.02% digitonin for 10 min at 4 °C and were further frozen and thawed 3 times to rupture the mitochondrial membrane. Then, the samples were centrifuged at 12,000× *g* for 10 min.

The substantia nigra tissue of the rats was collected, transferred to Tris-HCl buffer containing a protease inhibitor, and homogenated. The samples were frozen and thawed 3 times. The homogenate was centrifuged at 12,000× *g* at 4 °C for 10 min.

The supernatant of the samples was quantified using the BCA Protein Assay Kit before further experiments.

### 2.10. Colorimetric Ferrozine-Assay for the Quantitation of Iron

Ferrous iron was measured by the colorimetric ferrozine assay. The samples were incubated with 0.5 mM ferrozine and 1.5 M sodium acetate for 30 min at 37 °C, then OD_562_ was measured.

To further release iron from ferritin, a buffer containing 10% trichloroacetic acid and 10% hydrochloric acid was added to the samples at a volume ratio of 1:1. Then, the samples were heated at 95 °C for 20 min. After centrifugation at 12,000× *g* for 5 min, the supernatant was added with 0.5 mM ferrozine, 1.5 M sodium acetate and 10 mM ascorbate, then OD_562_ was measured.

### 2.11. Measurement of TBARS, BH_4_ and CoQ_10_

Then, 200 μL of the sample lysate and 120 μL of 1% 2-thiobarbital solution were mixed and then heated at 98 °C for 60 min. After centrifugation at 12,000× *g* for 10 min, the OD_562_ of the 200 μL supernatant was measured.

The BH_4_ and CoQ_10_ contents were measured using ELISA kits according to the corresponding instructions.

### 2.12. Western Blot

The brain tissue and cell lysates were prepared according to the standard protocol using RIPA buffer. Equal amounts (20 μg) of total protein were separated by 12% SDS-PAGE and then transferred to PVDF membranes. Membranes with proteins were subjected to blocking, washing, incubation with antibodies, and finally, detection with enhanced chemiluminescence. β-actin was used as the loading control.

### 2.13. Statistical Analysis

One-way ANOVA (Fisher’s LSD test) was performed using Graphpad Prism version 7.0 (GraphPad, La Jolla, CA, USA).

## 3. Results

### 3.1. Baicalein Is Predicted to Regulate ACO1 Based on Network Pharmacology

**Figure 1:**
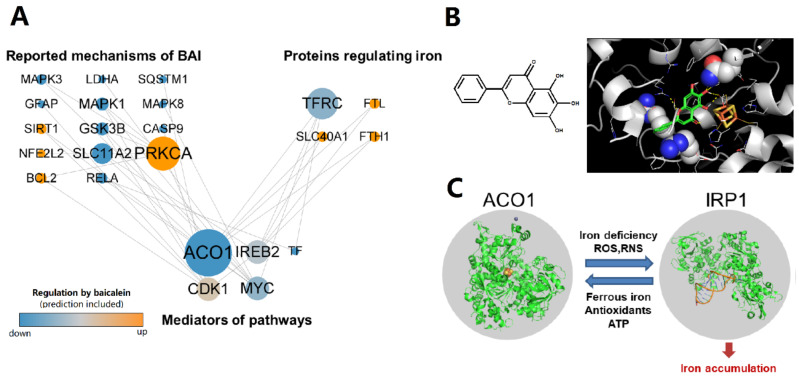
The regulation of ACO1 is a potential mechanism of baicalein against iron accumulation. (**A**) The regulation network from the reported mechanisms of baicalein to the proteins that regulate intracellular iron. The reported and predictive regulation of proteins by baicalein was colored orange (up-regulated) or blue (down-regulated). The size of nodes is positively correlated with the number of pathways that the protein participates in. (**B**) The chemical structure of baicalein and the molecular docking result that baicalein binds to ACO1. The substrate binding site of ACO1 is shown as spheres. The iron–sulfur cluster was colored yellow (sulfur) and orange (iron). (**C**) IRP1 can be activated from ACO1 to promote intracellular iron accumulation.

The regulation network of baicalein shows that ACO1 is a key node in the mechanisms wherein baicalein inhibits intracellular iron accumulation (Figure 1A). Molecular docking reveals that baicalein could bind near the iron–sulfur cluster of ACO1, which is the active site of ACO1 and the switch of ACO1 to IRP1 (Figure 1B). The docking score of baicalein to ACO1 is −4.1 kcal/mol. IRP1 is activated under oxidative stress and promotes intracellular iron accumulation (Figure 1C). These results suggest that baicalein could regulate ACO1.

### 3.2. Baicalein Protected ACO1 Activity against Oxidative Stress Induced by Hydrogen Peroxide and Sodium Nitroprusside

**Figure 2:**
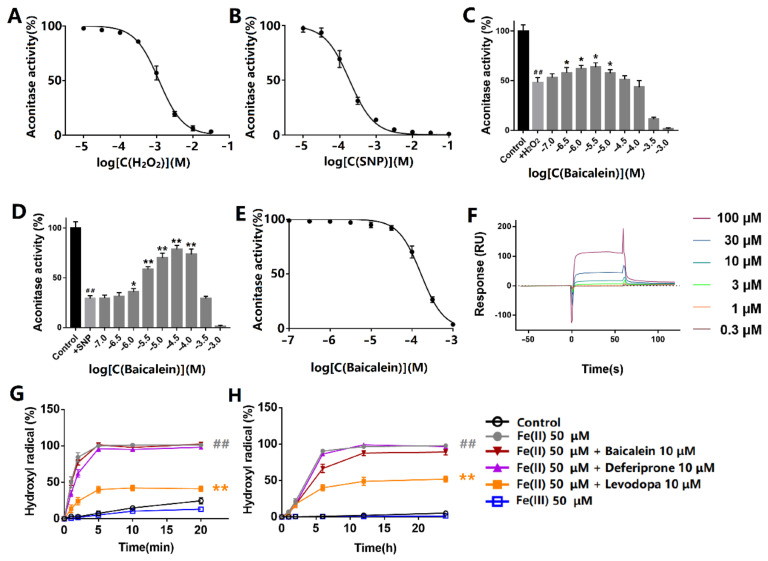
Effects of baicalein on ACO1 and ferrous iron-induced generation of hydroxyl radicals. (**A**,**B**) Effects of (**A**) hydrogen peroxide and (**B**) sodium nitroprusside on ACO1 activity. (**C**,**D**) The effect of baicalein on ACO1 activity treated with (**C**) 1 mM hydrogen peroxide or (**D**) 1 mM sodium nitroprusside. SNP: sodium nitroprusside. (**E**) The effect of baicalein on ACO1 activity. (**F**) The response units that baicalein binds to ACO1 by SPR assay. (**G**) Hydroxyl radical generated by iron and hydrogen peroxide. (**H**) Hydroxyl radical generated by iron and dissolved oxygen. The data are expressed as Mean ± SD (*n* = 3); ## *p* < 0.01 vs. control; * *p* < 0.05, ** *p* < 0.01 vs. model.

The effects of hydrogen peroxide and sodium nitroprusside on the aconitase activity were first evaluated. The protein was incubated with hydrogen peroxide or sodium nitroprusside at room temperature for 30 min and exposed to light. Hydrogen peroxide inhibited ACO1 activity with the IC_50_ at 1.26 mM (Figure 2A). Sodium nitroprusside inhibited ACO1 activity with the IC_50_ at 198 μM (Figure 2B). Baicalein (0.3–10 μM) protected ACO1 activity from hydrogen peroxide (Figure 2C). Additionally, baicalein (1–100 μM) significantly protected ACO1 activity from sodium nitroprusside (Figure 2D). However, baicalein incubation for 10 min inhibited ACO1 activity (Figure 2E) in the absence of hydrogen peroxide or sodium nitroprusside. The IC_50_ of baicalein was 166 μM.

The results of the SPR assay showed that baicalein could bind to ACO1 with the binding constant of Kd = 1.98 × 10^−4^ M (Figure 2F).

Oxidants release ferrous iron from iron–sulfur clusters, which induces the generation of hydroxyl radicals via the Fenton reaction. The results of hydroxyl radicals generated in the Fenton reaction are shown in Figure 2G. The hydroxyl radicals were generated immediately after the addition of hydrogen peroxide. Baicalein significantly decreased hydroxyl radical production, whereas deferiprone and levodopa did not show significant inhibitory effects. Apart from hydrogen peroxide, dissolved oxygen can also lead to the generation of hydroxyl radicals in the presence of ferrous iron (Figure 2H). Baicalein also inhibited the generation of hydroxyl radicals. However, deferiprone and levodopa had little effect on the production of hydroxyl radicals. Ferrous iron, rather than ferric iron, led to the production of hydroxyl radicals.

### 3.3. Baicalein Increased ACO1 Activity and Decreased IRP1 Levels in SH-SY5Y Cells Treated with Rotenone

**Figure 3:**
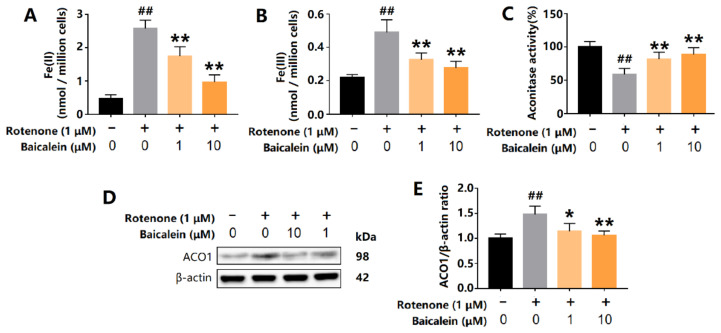
Effects of baicalein on intracellular iron content, ACO1 activity and ACO1 (and IRP1) protein level in SH-SY5Y cells treated with rotenone. (**A**) Intracellular ferrous iron. (**B**) Intracellular ferric iron. (**C**) Aconitase activity. (**D**,**E**) The expression of ACO1 (and IRP1) protein. The results are expressed as the relative level of protein compared with β-actin. Data are presented as Mean ± SD, *n* = 3. ## *p* < 0.01 vs. control; * *p* < 0.05, ** *p* < 0.01 vs. model.

The oxidative stress in SH-SY5Y cells was induced by rotenone, a complex I inhibitor. Rotenone significantly increased the content of intracellular ferrous and ferric iron. However, baicalein decreased intracellular levels of ferrous and ferric iron (Figure 3A,B). The total levels of ACO1 and IRP1 were measured using Western Blot. Rotenone increased the expression of ACO1 (and IRP1) in SH-SY5Y cells and was accompanied by a decrease in ACO1 activity, indicating that IRP1 was overexpressed and activated. Baicalein increased the ACO1 activity and also decreased the protein level of ACO1 and IRP1 (Figure 3C–E). These results reveal that baicalein inhibited the conversion of ACO1 to IRP1 in SH-SY5Y cells treated with rotenone.

### 3.4. Baicalein Improved Behavioral Impairments and Increased TH-Positive Cells in the Substantia Nigra of Rotenone-Induced PD Rats

**Figure 4:**
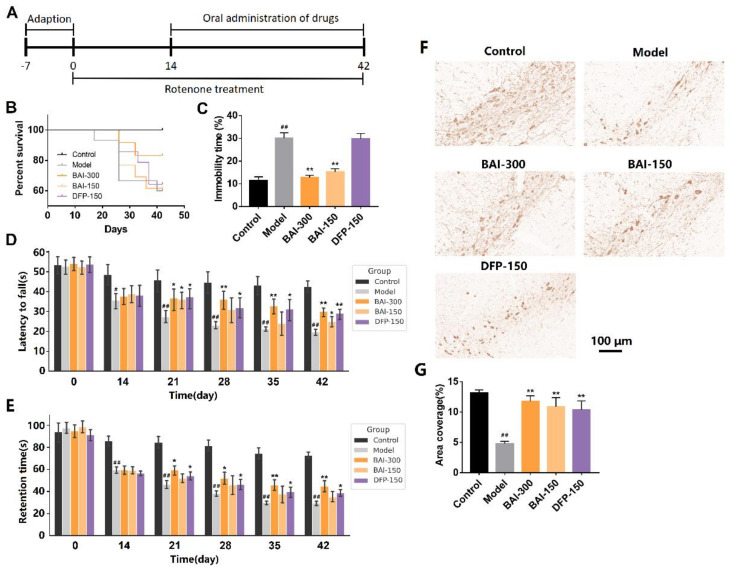
(**A**) Animal experiment schedule. In the rotenone-induced PD rats, (**B**) the survival function, (**C**) forced-swimming test performance, (**D**) rotarod test performance and (**E**) inclined plane test performance of rats in each group. (**F**) TH immunohistochemistry in the substantia nigra in the rotenone-induced PD rats. (**G**) Area coverage of TH immunohistochemistry in the substantia nigra. Data are presented as Mean ± SEM. Control: *n* = 10; Model: *n* = 9; BAI-300: *n* = 10; BAI-150: *n* = 8; DFP-150: *n* = 9. # *p* <0.05, ## *p* < 0.01 vs. control; * *p* < 0.05, ** *p* < 0.01 vs. model.

The PD rats induced with rotenone showed characteristics such as increased mortality, behavioral impairments, and the loss of dopaminergic neurons. Baicalein (300 mg/kg, q.d.) increased the survival rate of PD rats (Figure 4B). Depressive rats show immobility in forced-swimming tests, rather than jumping and swimming. Compared with the model group, baicalein (300 mg/kg, q.d. and 150 mg/kg, q.d.) significantly reduced the immobility time of the rats after entering the water (*p* < 0.01, Figure 4C). In the rotarod test, baicalein (300 mg/kg, q.d. and 150 mg/kg, q.d.) increased the time of rats staying on the rotarod (*p* < 0.01, Figure 4D). Baicalein also increased the retention time of the rats on the inclined plane (*p* < 0.01, Figure 4E). Baicalein (300 mg/kg, q.d. and 150 mg/kg, q.d.) increased TH-positive neurons, although not complete, in the substantia nigra of PD rats (Figure 4F,G).

### 3.5. Baicalein Inhibited the Activation of IRP1 and Alleviated Iron Accumulation in the Substantia Nigra of PD Rats Induced by Rotenone

**Figure 5:**
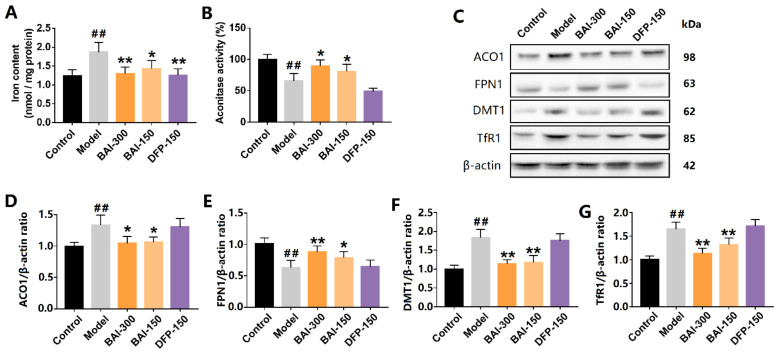
Baicalein inhibited IRP1 activation and iron accumulation in the substantia nigra of rotenone-induced PD rats. (**A**) Iron content in the substantia nigra. (**B**) Aconitase activity. (**C**–**G**) ACO1(and IRP1) and its iron-regulating downstream proteins FPN1, DMT1, and TfR1. All data are presented as Mean ± SEM, *n* = 3. ## *p* < 0.01 vs. control; * *p* < 0.05, ** *p* < 0.01 vs. model.

Consistent with the results from the SH-SY5Y cells, baicalein significantly alleviated iron accumulation in the substantia nigra of the PD model, and so did deferiprone (Figure 5A). Rotenone increased the expression of ACO1 (and IRP1) protein and decreased ACO1 activity, indicating the increased activation of IRP1. The activation of IRP1 was further confirmed by the results of the downstream proteins. DMT1 and TfR1 expression were increased in the substantia nigra of the PD model. Accordingly, FPN1 expression was decreased. DMT1 and TfR1 are the main mediators of iron import, while FPN1 is the only protein by which iron is exported from cells. Baicalein (300 mg/kg, q.d. and 150 mg/kg, q.d.) significantly inhibited ACO1 activity (Figure 5B). Further, baicalein decreased the expression of DMT1 and TfR1 and increased the expression of FPN1 (Figure 5C–G). Deferiprone, however, did not show a similar effect to that of baicalein. Deferiprone did not protect ACO1 activity from rotenone-induced oxidative stress, nor did deferiprone regulate IRP1, DMT1, TfR1, or FPN1.

### 3.6. Baicalein Inhibited Rotenone-Induced Lipid Peroxidation in the Substantia Nigra of PD Rats

**Figure 6:**
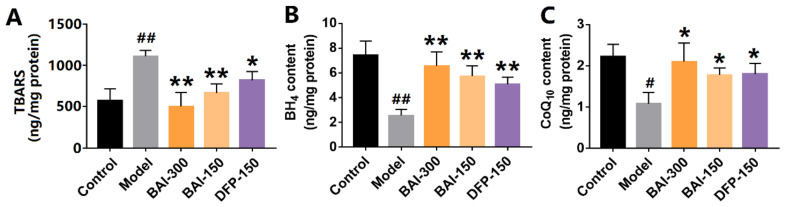
The metabolites on lipid peroxidation in the substantia nigra of rotenone-induced PD rats. (**A**) TBARS contents. (**B**) Tetrahydrobiopterin contents. (**C**) Coenzyme Q10 contents. The data are expressed as Mean ± SEM, *n* = 3; # *p* <0.05, ## *p* < 0.01 vs. control; * *p* < 0.05, ** *p* < 0.01 vs. model.

Iron overload and lipid peroxidation are key features of ferroptosis. Lipid peroxidation was measured using TBARS assay. The content of TBARS in the substantia nigra of PD rats was significantly increased by rotenone, whereas baicalein decreased the level of TBARS (Figure 6A). Baicalein also increased the levels of BH_4_ and CoQ_10_, the antioxidants against lipid peroxidation (Figure 6B,C), respectively. These results indicate that baicalein alleviated the iron accumulation and lipid peroxidation induced by rotenone.

## 4. Discussion

Iron-induced oxidative injury is a potential pathological mechanism of PD. Firstly, labile iron promotes dopamine oxidation, generating toxic products such as the complex I inhibitor, aminochrome [35,36]. Secondly, iron induces free radical damage to mitochondria via the Fenton reaction [37,38]. Thirdly, iron induces the aggregation of α-synuclein through ligand bridge interactions [39]. Additionally, iron is associated with inflammatory factors such as TNF-α and IL-6 [40].

IRP1 is activated upon iron depletion or oxidative stress, promoting intracellular iron accumulation [41]. IRP1 can bind to iron-responsive elements (IRE) in certain mRNAs. Then, IRP1 promotes or inhibits the expression of mRNAs depending on whether the IRE is in the 3′- or 5′ untranslated regions. IRP1 binds to the 3′ IRE of TfR1 and DMT1 to stabilize their mRNA and increase their half-life [42]. TfR1 promotes iron uptake from transferrin to cells [43]. DMT1 transports ferrous iron and many other divalent metal ions [44]. On the other hand, IRP1 binds to the 5′ IRE of FPN1 to inhibit mRNA translation [45]. FPN1 is involved in the iron release from cells [46]. Rotenone, an inhibitor of complex I, inhibits the electron transfer from the iron–sulfur centers of complex I to CoQ_10_ and leads to the formation of reactive oxygen species (ROS) [47]. The balance between ACO1 and IRP1 is disturbed in 6-OHDA and rotenone-induced PD animal and cellular models [9,48]. The activation of IRP1 plays a key role in rotenone-induced SH-SY5Y cell death [49].

**Figure 7:**
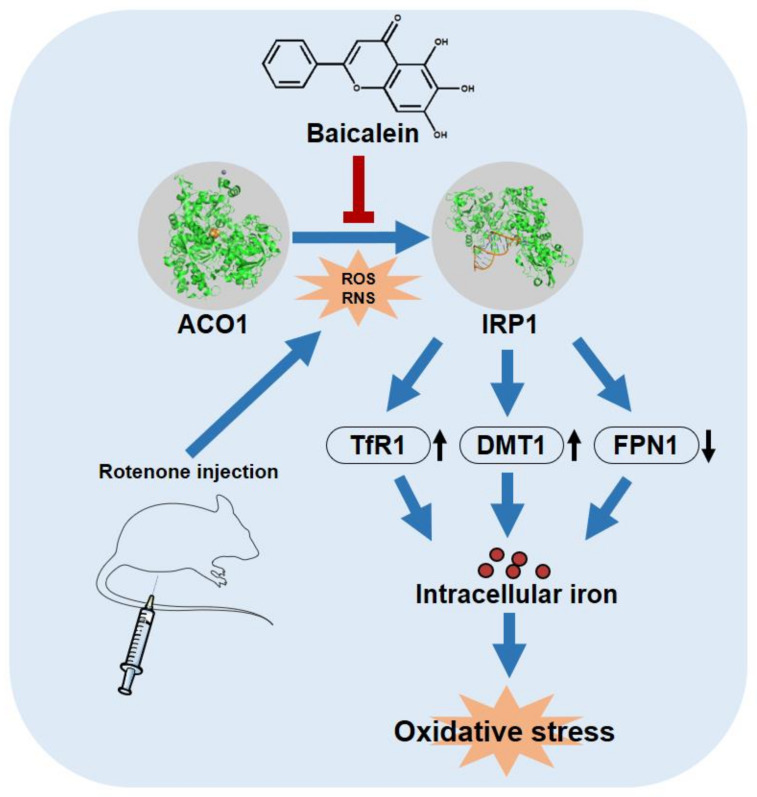
Baicalein attenuated iron accumulation by inhibiting the activation of IRP1 from ACO1. Meanwhile, baicalein inhibited iron-induced oxidative stress in the brain of rotenone-induced PD rats. The protein structure of ACO1 and IRP1 are from PDB: 2B3X and PDB: 3SNP, respectively.

We reveal that baicalein inhibited the rotenone-induced activation of IRP1 and further inhibited iron accumulation in the substantia nigra of PD rats. Baicalein decreased the expression of TfR1 and DMT1 and increased the expression of FPN1, possibly by inhibiting IRP1 activation (Figure 7). The protective effect of baicalein may be due to its role as an antioxidant rather than merely as an iron chelator. Baicalein has a Kd value of 1.98 × 10^−4^ M for binding to ACO1 and an IC_50_ of 1.66 × 10^−4^ M for inhibiting ACO1 activity. The proximity of these two values suggests that baicalein could bind near the active site of ACO1 and then affect ACO1 activity. The iron–sulfur cluster of ACO1 is also at its active site, and the binding of baicalein to ACO1 may further protect the iron–sulfur cluster from ROS and RNS. Meanwhile, iron is thought to induce oxidative stress by the liberation of oxygen free radicals from hydrogen peroxide. At a concentration much lower than iron, baicalein, rather than the iron chelator, deferiprone, strongly inhibited iron-induced hydroxyl radicals. The evidence suggests that baicalein is more than an iron chelator. Still, the ability of iron chelation and ACO1 binding may contribute to the protective effects of baicalein. Iron chelation therapy deferiprone is the positive control in this study. Deferiprone chelates ferric iron to promote iron excretion from cells [50]. We report that deferiprone could not inhibit IRP1 activation. A previous study shows that mitochondrial iron is chelated by deferiprone, inhibiting aconitase activity [51]. As a result, baicalein differs from deferiprone in the mechanisms of alleviating iron accumulation.

Simultaneously, baicalein increased the contents of endogenous antioxidants BH_4_ and CoQ_10_, both of which can inhibit lipid peroxidation and counteract ferroptosis [52,53]. In addition, BH_4_ is an essential cofactor of TH, and BH_4_ deficiency decreases the levels of TH and dopamine [54]. CoQ_10_ is the electron acceptor for complex I and complex II, whose levels correlate with the activity of mitochondria in patients with PD [55]. The oral administration of CoQ_10_ shows therapeutic effects in MPTP-, 6-OHDA-, and rotenone-induced PD models [56,57,58]. Baicalein increased the contents of BH_4_ and CoQ_10_, possibly through the inhibition of iron accumulation and iron-induced oxidative stress.

## 5. Conclusions

This study reveals the effects of baicalein on ACO1 and its ability to inhibit iron accumulation in PD models. Baicalein protected the aconitase activity and inhibited IRP1 activation by oxidative stress in molecular, cellular, and animal experiments.

## Data Availability

The data are contained within the article.
